# Focused particle beam-induced processing

**DOI:** 10.3762/bjnano.6.191

**Published:** 2015-09-09

**Authors:** Michael Huth, Armin Gölzhäuser

**Affiliations:** 1 Goethe Universität, Physikalisches Institut, Max-von-Laue-Str. 1, D-60438 Frankfurt am Main, Germany; 2 Universität Bielefeld, Fakultät für Physik, Universitätsstr. 25, D-33615 Bielefeld, Germany

In light of the success of 3D printing using fused-deposition modeling or higher-resolution variants with lasers applicable to polymers and metals, one may wonder whether an analogous approach exists on the nanometer scale. Indeed it does. With the aid of focused particle beam-induced deposition (FPBID) it is possible to create solid-state structures on the nanoscale. However, in contrast with large-scale 3D printing of plastic or metallic structures, FPBID provides nanomaterials with a wealth of interesting electronic, optical and magnetic properties. Due to this, focused electron beam-induced deposition (FEBID) has experienced a rapid expansion in the breadth of its application fields over the last 10 years.

In FEBID, a highly focused electron beam, in most cases provided by a scanning electron microscope, is raster-scanned over a substrate surface on which an adsorbed precursor layer is sustained by a precursor gas injection system. As the primary (as well as the secondary and backscattered) electrons of the focused beam dissociate the precursor adsorbate, a permanent deposit is formed. Depending on the precursor and other process parameters, amorphous, nanogranular, nanocrystalline or polycrystalline nanostructures are obtained. Their position and shape (i.e., also in 3D) are fully controlled by the precise movements of the electron beam. Under optimized conditions, the intrinsic resolution limit of FEBID is below 5 nm.

In this Thematic Series, the FEBID process is considered from different perspectives. The current knowledge of the most relevant dissociation channels at play in FEBID are reviewed in the article by Rachel Thormann and coworkers [1]. FEBID-specific continuum modeling approaches are elucidated in a review article by Milos Toth and collaborators [2]. An aspect thus far not considered in electron beam-induced growth is the role of surface excitations, as pointed out in the article by Francesc Salvat-Pujol et al. [3]. Recently, in an effort towards obtaining pure metal nanostructures, the postprocessing of FEBID structures has become an active field of research. This is covered in this Thematic Series by Brett Lewis and coworkers with a focus on platinum [4]. The postprocessing of copper-based FEBID structures by vacuum annealing is presented by Aleksandra Szkudlarek and collaborators [5]. Additionally, high electric current densities can be used to induce structural changes in suspended nanowires, as is shown for cobalt deposits in the article by Gian Carlo Gazzadi and Stefano Frabboni [6]. This leads into the important application field of magnetic nanostructures obtained by FEBID. Luis Rodríguez and coworkers present a detailed study on the influence of shape anisotropy and surface oxidation on the magnetization reversal of thin, iron nanowires [7]. In the article by Oleksandr Dobrovolskiy and colleagues [8], different postgrowth purification treatments for platinum and cobalt FEBID structures are employed to fine-tune the magnetic properties of heterostructures. A novel application of electron beam-induced deposition of amorphous carbon for the patterning of gold nanoparticle structures is introduced by Takahiro Noriki and coworkers [9]. The resolution limiting aspects are covered in the article by Roland Schmied and collaborators concerning fundamental edge-broadening effects in FEBID [10].

A more recent development that may help to alleviate the resolution-limiting issues in FEBID on solid substrates is the employment of helium ion microscopy (HIM). In its current development stage, HIM is mainly used for imaging applications, providing enhanced contrast for surface features as compared to scanning electron microscopy. Along this direction, Yuri Petrov and Oleg Vyvenko have exploited reflected helium ions for high-resolution imaging with “chemical contrast” [11]. Hongzhou Zhang and coworkers have utilized a focused helium ion beam to modify and mill thin silicon foils [12], which constitutes pioneering work in HIM towards nanofabrication. Along the same path, Gregor Hlawacek, Bene Poelsema and coworkers focused on the interaction of helium ions with metal surfaces (gold in particular) [13–15]. In a series of three distinct articles, they concentrate on ion channeling, crystal mapping, and finally, ion-induced modification of the gold surface. A novel route towards focused particle beam-induced processing (FPBIP) with HIM is paved by Xianghui Zhang and coworkers [16]. They used focused helium ions to perform the controlled modification of materials in monomolecular organic films. Here, ion exposure induced 2D polymerization that is a basis for the creation of ultrathin nanomembranes. Finally, André Beyer and coworkers show impressive HIM images of ultrathin carbon nanomembranes [17], which is a clear indication of the potential of the bourgeoning fields of helium ion microscopy and lithography towards nanofabrication with focused charged particles.

The idea for this Thematic Series arose in conjunction with the 5th International Workshop on Focused Electron Beam Induced Processing (FEBIP2014) held at the Physics Department of the Goethe University in Frankfurt am Main in July 2014, which brought together renowned experts in the field. A selection of the more than 80 contributions to the workshop are presented in this Thematic Series in the form of original research articles reflecting recent advances in FPBIP.

We would like to express our sincere gratitude to all the authors for contributing their excellent work to this Thematic Series. We would also like to give special thanks to all referees for their timely and highly valuable reports. This helped tremendously to keep the publication times short and thus attractive for all contributors. Last but not least, we thank the team at the Beilstein-Institut for their continuous support. In particular, we would like to acknowledge the open access policy of the Beilstein Journal of Nanotechnology, which guarantees that this Thematic Series, together with a wealth of other articles, is freely available to the community.

Michael Huth and Armin Gölzhäuser

Frankfurt am Main and Bielefeld, August 2015
